# Musculoskeletal sequelae in patients with obstetric fistula – a case–control study

**DOI:** 10.1186/s12905-014-0136-3

**Published:** 2014-11-08

**Authors:** Merete Kolberg Tennfjord, Mulu Muleta, Torvid Kiserud

**Affiliations:** Centre for International Health, University of Bergen, Bergen, Norway; Obstetric Fistula Wing, Gondar University Hospital, University of Gondar, Gondar, Ethiopia; Research Group for Pregnancy, Fetal Development and Birth, Department of Clinical Science, University of Bergen, Bergen, Norway; Department of Obstetrics and Gynecology, Haukeland University Hospital, Bergen, Norway

**Keywords:** Obstetric fistula, Musculoskeletal injuries, Muscle strength, Joint range of motion, Foot drop, Pain

## Abstract

**Background:**

Obstetric fistula is essentially a result of pelvic injury caused by prolonged obstructed labour. Foot drop and walking difficulties in some of these women signify that the injury may extend beyond the loss of tissue that led to the fistula. However, these aspects of the pelvic injury are scarcely addressed in the literature. Here we specifically aimed at assessing musculoskeletal function in women with obstetric fistula to appreciate the extent of the sequelae of their pelvic injury.

**Methods:**

This case–control study compared 70 patients with obstetric fistula with 100 controls matched for age and years since delivery. The following was recorded: height, weight, past and present walking difficulties, pain, muscle strength and joint range of motion, circumference and reflexes. Differences between groups were analysed using independent sample t-test and chi-square test for independence.

**Results:**

A history of leg pain was more common among cases compared to controls, 20% versus 7% (p = 0.02), and 29% of the cases had difficulties walking following the injuring delivery compared to none of the controls (p ≤ 0.001). Of these, four women reported spontaneous recovery. Cases had 7° less range of motion in ankle dorsal flexion (95%CI: −8.1, −4.8), 8° less ankle plantar flexion (95%CI: −10.6, −6.5), 12° less knee flexion (95%CI: −14.1, −8.9), and 4° less knee extension (95%CI: 2.9, 5.0) compared to controls. Twelve % of the cases had lower ankle dorsal flexion strength (p = 0.009). Foot drop was present in three (4.3%) compared with none among controls. Women with fistula had 4° greater movement in hip extension (95%CI: −5.9, −3.1), 2° greater hip lateral rotation (95%CI: 0.7, 3.3) and 9° greater hip abduction (95%CI: 6.4, 10.7). Twelve % of the cases had stronger medial rotation in the hip (p = 0.04), 20% had stronger hip lateral rotation (p ≤ 0.001), 29% had stronger hip extension (p ≤ 0.001), and 15% had stronger hip abduction (p = 0.04) than controls.

**Conclusions:**

Women with obstetric fistula commonly experienced walking difficulties after the delivery, had often leg pain and reduced function in the ankle and knee joints that may have been compensated by increased motion and strength in the hip.

**Electronic supplementary material:**

The online version of this article (doi:10.1186/s12905-014-0136-3) contains supplementary material, which is available to authorized users.

## Background

Obstetric fistula is an abnormal opening between the bladder and the vagina or the rectum and the vagina, resulting in continuous and unremitting urinary or fecal incontinence [1]. Women with obstetric fistula often suffer additional urologic, gynaecologic, gastrointestinal, musculoskeletal, neurologic, or dermatologic complications, foetal loss and social disruption as a consequence of the fistula or a direct cause of the obstetric damage [1-3]. The majority of the injuries to the pelvic tissues are results of the prolonged obstruction of labour with corresponding longstanding compression and stretching of the soft tissue of the bladder, vagina and rectum [4]. Neurological injuries may arise due to compression of the foetal head on the lumbosacral nerve trunk in the pelvis or as an injury to the peroneal nerve behind the fibular head due to prolonged squatting [3].

Peroneal nerve injury, resulting in leg weakness and foot drop has commonly been found associated with obstetric fistula. Studies report on foot drop in 20-30% of these women [2,5,6]. Walking difficulties have been reported in 1.5% of primiparous (95%CI: 1.47, 1.53) and 0.8% in multiparous fistula patients (95%CI: 0.6, 1.0) [7]. Most such studies have aimed at assessing the fistula with less focus on the neural and musculoskeletal signs. We believe that by addressing musculoskeletal function beyond that of the obvious foot drop in patients with obstetric fistula, we may get a more comprehensive understanding of the pelvic injury caused by the prolonged obstructed labour.

The specific objective of the present study was to identify musculoskeletal impairments in women with obstetric fistula.

## Methods

### Study design

This case–control study was conducted during a period of 11 months in 2010–11 at Gondar University Hospital, Gondar, Ethiopia. The study was approved by the Regional Committee for Medical and Health Research Ethics, Western-Norway (REK Vest 2010/503) and the ethics committee of Gondar University, Ethiopia (RCS/05/540/2010), and carried out in agreement with the Helsinki declaration (2008). The study was also granted permission to examine fistula patients in three rural outpatient departments run by the non-governmental organisation Intrahealth. Eligible patients were informed about the study by an Amharic speaking health professional. They gave their informed consent either by signature or fingerprint before participating in the study. This study followed the Strobe reporting guidelines (Additional file 1).

### Participants

Fistula cases were recruited from the hospital area, i.e. gynaecological ward and gynaecological outpatient department, in addition to three fistula units in the same region. The fistula patients presented themselves to the hospital in Gondar or were invited through information campaigns. An inclusion criterion was that the woman had a verified fistula following a child birth.

Controls were recruited among women attending the hospital out-patient departments, but were excluded if they attended the hospital because of trauma or surgery to the lower extremities or had had neurological injuries of their lower extremities unrelated to obstetric history. The controls were recruited provided they came from a rural area and matched for age (±5 years) and years since delivery (±5 years).

### Outcomes

Prior to the study, there was a preparatory period to assess feasibility and test measurement procedures in 12 participants carried out by the two physical therapists involved in the study. A protocol was prepared and the test procedures were conducted twice with a few days in between in each volunteer to ensure reproducibility. The preparatory period was conducted in Norway by the main investigator and in Gondar by the second investigator. It was not possible for the investigators to do simultaneous testing on the same women. Due to time constrains a walk test and sensory testing were not carried out. A history of the participating women was taken and translated by an Amharic speaking health professional. Information was noted on walking difficulties before the delivery, immediately after the delivery and at present. Difficulties with activities of daily life (ADL) were noted if it was related to walking when taking care of the house and the children, cooking, cleaning etc. The information on walking and ADL was registered as yes or no. Additionally the following were registered: time since labour and childbirth that caused the fistula (or the last labour for the control group), mode of the delivery, foetal outcome, sex of neonate and pain in the lower extremities following the delivery. The latter was drawn from a figure in which the women were asked to mark if they had pain somewhere in the body. Information from hospital records included type of fistula.

The participants removed heavy clothing and shoes, and underwear was offered. The measurements were standardised for verbal instruction, participant’s positioning, point of measurement, fixation and stabilisation of the lower extremities, and recording of data. Both legs were measured. Each examination took approximately 60 minutes.

### Anthropometry

Weight was measured twice on a digital scale and recorded to the nearest 0.1 Kilogram (Kg). Height*,* upper arm, leg and ankle circumferences were measured twice with a non-stretch tape and the mean recorded to the closest 0.1 centimetres (cm). Height was measured with the participant standing with the back against a wall and a mark was placed on the wall, in a horizontal line from the top of the participant’s head. Upper arm circumference was measured in a horizontal direction from the axilla, leg circumference 15 cm distal to the distal point of the patella and ankle circumference just proximal to the malleoli.

### Joint range of motion

Testing of passive joint range of motion was adapted from Norkin and White [8]. Both legs were measured twice with a goniometer and the highest score was registered. Bony landmarks were first identified before the goniometer placement. Six movements were tested for the hip: flexion, extension, abduction, adduction, internal and external rotation. For the knee and ankle, flexion and extension were measured.

### Muscle strength testing

Muscle strength of hip, knee and ankle was examined according to Kendall et al. [9]. The joint to be tested was positioned in full or close to full range of motion and graded as follows: “grade 0: no contraction felt or seen in the muscle, grade 1: tendon becomes prominent or feeble contraction felt in the muscle with no visible movement, grade 2: not able to hold test position against gravity, grade 3: holds test position in the antigravity position (no pressure added), grade 4: holds test position against moderate pressure in the antigravity position, grade 5: holds test position against strong pressure in the antigravity position” [9]. Ankle plantar flexion was tested standing with support and the participant was asked to rise on toes ten times. Knee flexion strength was tested alternatively supine with gravity if prone against gravity was not comfortable.

### Reflexes

Patellar and Achilles reflexes were tested in order to document neurological deficits and was recorded as visible or missing [10,11]. The participants had their eyes closed and were encouraged to relax. The tendon was tapped several times to facilitate the reflex.

### Statistical analysis

The statistical analysis was performed in SPSS version 16.0. Power calculation was based on the assumption that prevalence of musculoskeletal deficits in women with obstetric fistula was 10% and in the control group 1%. With the power of 80% and 95%CI, 100 cases and 100 controls were planned included. Population characteristics were presented with mean and SD or median and range. Results from muscle strength testing were presented dichotomised, i.e. grade 5 if maximum resistance could be tolerated for three seconds, and <5 if maximum resistance could not be tolerated for three seconds or less, or if no resistance was applied. This was done to ensure meaningful statistical power in the subgroups. Differences in groups of muscle strength, pain, reflexes, walking difficulties and difficulties with ADL were assessed using chi-square test for independence. Differences in groups regarding joint range of motion and circumference were assessed with independent sample t-test. Left and right leg in both groups were pooled when analysing joint range of motion, muscle strength, reflexes and circumference, thus increasing the statistical power of the groups. Incomplete or missing data were included in the analysis and are listed in the tables were appropriate. P-values <0.05 were considered statistically significant.

## Results

### Socio-demographic characteristics

Due to time constrain only 70 of the intended 100 fistula cases were recruited within the allocated project period. Population characteristics of the 70 fistula cases and 100 controls included in this study are presented in Table 1. There was a significant difference between cases and controls for weight, 44.9 Kg, (SD 6.8) versus 48.7 (SD 8.2) (95%CI: 0.9, 5.5), and for height, 151.6 cm (SD 7.4) versus 156.7 (SD 5.6) (95%CI: −7.1, −3.0), respectively, but no difference in body mass index, 19 (SD 2.4) versus 19.31 (SD 3.3) (95%CI: −1.2, 0.6), respectively. Mean age at the time of the childbirth that caused the fistula was 19 years for primiparous and 29 years for multiparous (95%CI: −12.3, −5.6). Fifty two (78.8%) had developed a vesico-vaginal fistula, 10 (15.2%) urethro-vaginal fistula, and four (6.1%) recto-vaginal fistula. One case (1.5%) had combined vesico-vaginal and urethro-vaginal fistula*.* Obstetric data was missing for four cases.

**Table 1:** **Background characteristics for fistula cases and controls**

	**Cases (n = 70)**	**Range**	**Controls (n = 100)**	**Range**	**95%CI of the difference**
Age (years)†	31.5 (SD 12.1)	15.0-63.0	31.7 (SD 8.1)	20.0-65.0	−3.6, 2.8
Weight (kg)†	44.9 (SD 6.8)	30.7-64.8	48.7 (SD 8.2)	29.8-72.5	**−6.1, −1.4**
Height (cm)	151.6 (SD 7.4)	132.5-172.0	156.7 (SD 5.6)	140.7-173.0	**−7.1, −3.1**
BMI (kg/m^2^)†	19.0 (SD 2.4)	13.0-25.0	19.3 (SD 3.3)	12.0-31.0	−1.2, 0.6
Duration of labour (days)††#	3.0	0.1-8.0	0.2	0.1-5.0	**<0.001**
Years since labour†#	4.0	0.1-36.0	4.0	0.2-30.0	0.77

*Presented as means with SD, range and 95%CI.*

*#Median presented with p-values.*

*Kg = kilograms, cm = centimetres, BMI = body mass index.*

*N = †Cases n = 69, ††Controls n = 96.*

*Bold numbers indicate statistical significance.*

The majority of the participants came from rural communities, i.e. 69 (98.6%) of the fistula cases and 95 (95%) of the controls (p = 0.4). Fifteen (21.4%) of the cases and 5 (5%) of the controls had caesarean delivery (p = 0.002). Thirty-three (47.1%) of the cases were primiparous versus 19 (19%) of the controls (p < 0.001). Among the fistula cases 34 (48.6%) delivered a male neonate, 16 (22.9%) delivered a female neonate and 20 (28.6%) said they didn’t know the sex of their neonate. In comparison, controls had a male delivery in 53 (53%), female delivery in 46 (46%) and 1 (1%) that didn’t know the sex of the neonate, a difference being in the female and unknown sex (p < 0.001). The fistula cases had had 19 (27.1%) live births while controls had 97 (97%) (p < 0.001). Three (16%) of the live born neonates in the fistula group died within the first week of life, and none in the control group.

### Musculoskeletal findings

Fistula cases complained more commonly that walking difficulties occurred after the delivery (Table 2). Four (5.7%) cases reported that their walking difficulties had recovered spontaneously, whereas 16 (22.8%) still had the problem. Two fistula cases (2.8%) and 16 (16%) controls had developed walking difficulties recently. One fistula case (1.4%) with walking difficulties before the index pregnancy had these difficulties at present and four (4%) of the controls. There was no difference concerning difficulties with ADL between the groups (Table 2). Fourteen (20%) of the cases reported more leg pain following the delivery compared with 7 (7%) of the controls (p = 0.02).

**Table 2:** **Difficulties walking and with activities of daily living (ADL) in fistula cases and controls**

	**Cases (n = 70)**	**Controls (n = 100)**	**P-value**
Walking difficulties before the index pregnancy	1.0 (1.4)	4.0 (4.0)	0.61
Walking difficulties after delivery	20.0 (28.6)	0 (0)	**<0.001**
Present walking difficulties	19.0 (27)	20.0 (20.0)	0.37
Difficulties with ADL before the index pregnancy	2.0 (2.9)	3.0 (3.0)	1.00
Present difficulties with ADL	15.0 (21.4)	22.0 (22.0)	1.00

*Presented as frequencies with (%) and p-values.*

*Bold numbers indicate statistical significance.*

The results of the clinical examination show left and right leg pooled, fistula cases n = 140, controls n = 200. Fistula cases had smaller mean upper arm circumference (25.1 versus 26.2 cm) (95%CI: −1.7, −1.4) and greater mean ankle circumference (21.1 versus 19.8 cm) (95%CI: 0.8, 1.8). Mean leg circumference was 28.1 cm in the fistula group and 28.5 cm among the controls and not significantly different (95%CI: −1.2, 0.2). Thirty (15.2%) controls had no visible Achilles reflex compared with 5 (3.6%) fistula cases (p = 0.001). Four (2.9%) of the cases versus 10 (5.0%) of the controls had no visible patella reflex, but this was not significant (p = 0.5). Results from testing of joint range of motion and muscle strength are presented in Tables 3 and 4, respectively. Four (5.7%) cases had no movement in passive ankle dorsal flexion, and in one case it was found bilaterally. Passive ankle dorsal flexion was the movement beyond the anatomic position which is with the foot positioned in 90° to the leg bones. We found right sided foot drop in three (4.2%) cases. Knee flexion strength was tested prone and against gravity in 130 out of 138 (94.0%) cases versus 136 (70%) out of 196 controls. The rest, 6% of cases and 30% of controls, was tested supine and with gravity.

**Table 3:** **Passive joint range of motion among fistula cases and controls**

	**N**	**Cases**	**N**	**Control**	**95%CI of the difference**
Hip medial rotation	140	41.7 (SD 6.3)	200	41.7 (SD 6.9)	−1.4, 1.4
Hip lateral rotation	140	38.2 (SD 5.9)	197	36.2 (SD 6.3)	**0.7, 3.3**
Hip flexion	140	112.1 (SD 7.8)	196	113.1 (SD 10.5)	−2.9, 1.0
Hip extension†	132	−17.4 (SD 6.6)	137	−12.9 (SD 4.9)	**−5.9, −3.1**
Hip abduction	137	32.6 (SD 10.3)	186	24.1 (SD 8.7)	**6.4, 10.7**
Hip adduction	140	24.5 (SD 4.6)	190	24.3 (SD 5.2)	−0.9, 1.1
Knee flexion	140	142.3 (SD 14.6)	198	153.8 (SD 6.1)	**−14.1, −8.9**
Knee extension†	140	−3.2 (SD 4.6)	198	−7.2 (SD 5.3)	**2.9, 5.0**
Ankle dorsal flexion	140	19.9 (SD 8.2)	198	26.4 (SD 6.9)	**−8.1, −4.8**
Ankle plantar flexion	140	45.8 (SD 10.1)	197	54.3 (SD 8.1)	**−10.6, −6.5**

*Presented as numbers, degrees with (SD) and 95%CI.*

*Left and right leg was pooled making a total of 140 cases and 200 controls. Variation in N reflects incomplete numbers of examinations among the participants.*

*†Joint movement below 0 indicates hyperextension of the joint.*

*Bold numbers indicate statistical significance.*

**Table 4:** **Muscle strength according to manual muscle testing; scale 1–5, 5 being the strongest**

	**Fistula cases**			**Control group**			**P-value**
	**N**	**Gr 5**	**Gr <5**	**N**	**Gr 5**	**Gr <5**	
Hip medial rotation	138	62 (44.9%)	76 (55.1%)	198	66 (33.3%)	132 (66.7%)	**0.04**
Hip lateral rotation	138	57 (41.3%)	81 (58.7%)	198	43 (21.7%)	155(78.3%)	**<0.001**
Hip flexion	138	60 (43.5%)	78 (56.5%)	200	68 (34%)	132 (66%)	0.10
Hip extension	120	40 (33.4%)	80 (66.7%)	113	5 (4.4%)	108 (95.6%)	**<0.001**
Hip abduction	138	54 (39.1%)	84 (60.9%)	198	47 (23.7%)	151 (76.3%)	**0.04**
Hip adduction	136	26 (19.1%)	110 (80.9%)	195	31 (15.9%)	164 (84.1%)	0.54
Knee flexion	138	49 (35.5%)	89 (64.5%)	196	29 (14.8%)	167 (85.2%)	**<0.001**
Knee extension	138	82 (59.4%)	56 (40.6%)	200	116 (58%)	84 (42%)	0.88
Ankle dorsal flexion	135	97 (71.9%)	38 (28.1%)	198	167 (84.3%)	31 (15.7%)	**0.009**
Ankle inversion	136	78 (57.4%)	58 (42.6%)	198	122 (61.6%)	76 (38.4%)	0.51
Ankle eversion	136	84 (61.8%)	52 (38.2%)	198	126 (63.6%)	72 (36.4%)	0.82
Ankle plantar flexion	140	112 (80%)	28 (20%)	182	160 (87.9%)	22 (12.1%)	0.07

*The results are dichotomised in grade5 or <5 and presented with numbers (%) and p-value for the difference between the fistula and control group. Left and right leg was pooled making a total of 140 cases and 200 controls. Variation in N reflects incomplete examination numbers among the participants.*

*Bold numbers indicate statistical significance.*

## Discussion

### Main findings

The present study showed that self-reported pain in the lower extremities and walking difficulties following the delivery were more common among fistula patients compared with controls. They had also lower joint range of motion and lower strength at the level of knee and ankle, and foot drop was found in three (4.2%) women with obstetric fistula. On the other hand side, these women were significantly stronger and had greater joint range of motion at the level of the hips, which suggests a compensatory adaptation.

### Interpretation of findings

Self-reporting is prone to bias. That may also be the case for reported pain and walking difficulties in our fistula patients. However, these findings are supported by previous reports [5], and have been described in Ethiopian population [7,12]. Muleta et al. [7] reported impaired walking in 127/8479 (1.5%) in primiparous fistula patients and 52/6449 (0.8%) in the multipara patient. The high number of primiparous women in the study group (one of the factors thought to increase the risk of developing neurological and musculoskeletal deficits [7]) may have skewed the result towards a larger difference. Although more cases reported difficulties in walking, difficulties with daily life activities were similar. This could be interpreted as that the walking problems were minor since ADL were not different from the controls. On the other hand side, the reduced Achilles reflex in the control group could possibly reflect their reasons for seeking medical help. One could therefore suggest that ADL in fistula cases were affected at the same level as patients otherwise attending the hospital.

Foot drop was found in three (4.2%) cases, which is lower than that reported by Arrowsmith et al. [2] (20%), Waaldijk and Elkins [5] (26%) and Williams [6] (30%). An explanation for the high number of women with foot drop found by Arrowsmith et al. [2] and Williams [6] could be a selection effect, the more severe cases being referred to their centre while the less extensive cases were operated locally. Secondly, the register had been based on informal surveys. In Nigeria Waaldijk and Elkins [5] used a similar scale to that used in the present study for grading muscle strength. Of their 211 patients with prior peroneal nerve trauma and a fistula of over two years duration, only 28 (13.3%) had persistent physical signs of nerve trauma. This could be a reasonable explanation for the few cases of foot drop found in our study where median time since the injuring labour was four years. Four (5.7%) cases reported that their walking difficulties had healed spontaneously. A possible development would be that the damaged neurons may not have been totally severed and the neurons recovered in the following months and years, earliest for the proximal portions of the lower extremity and latest for the ankle and foot function [13]. The low number of women presenting with foot drop in our study could also be a result of selection bias, as the cases examined were able to access the hospital, leaving the most injured at home. Foot drop was found on the right side, a finding seen in previous studies [5,13].

Women with obstetric fistula were significantly stronger in muscle groups related to the hip, which could, as mentioned above, reflect a compensatory adaption since they had both a decreased joint range of motion in the ankle joint and decreased strength in ankle dorsal flexion. The loss of dorsal flexion strength in the ankle forces the women to abduct more at the level of the hip to make the toes clear the ground (swing-out gate) or add extra flexion to the hip and knee to raise the foot (high-steppage gait) [11]. The increase in hip movement could also be explained similarly, but cultural issues might also play a part. The controls, some of them admitted for non-obstetric reasons, may have felt too exposed when undressing for the hip assessment.

Why we were not able to demonstrate diminished or absent Achilles reflex among the fistula cases could be due to the fact that we only registered visible versus non-visible tendon response and might have missed some information about more subtly decreased movements. Another explanation could be that testing of reflexes is not sensitive enough and should have been used in conjunction with other neurological measurements [14].

Long duration of labour, high numbers of stillbirths, lower weight and stature, more primiparous and young age at the time of delivery in our fistula group are in line with a previous study in the same population [7], and is supported by other studies [15,16]. However, Hilton and Ward [4] and Ezegwui and Nwogu-Ikojo [17] found higher age and more multipara with obstetric fistula compared to the present study. The study by Ezegwui and Nwogu-Ikojo [17] also reported on a higher rate of caesarean deliveries and shorter duration of labour as characteristics for their fistula patients probably reflecting ethnic and cultural variations, and possibly higher number of iatrogenic fistulas. The high rate of male neonates has also been pointed out previously as a documentation of cephalopelvic disproportion being a main cause of the prolonged labour in this condition [7,16]. However, almost 30% of the cases in our study reported unknown sex of their stillborn neonate. This makes our data of male neonates as a factor in obstructed labour inconclusive.

We found fistula cases to have a marginally but significantly smaller upper arm circumference. This was concordant with their lower weight and stature, and in line with a previous study [7]. In that study as in the present, body mass index was equal in fistula cases and controls suggesting that their body proportion and nutritional status were similar.

There were no combined vesico-vaginal and recto-vaginal fistulas, previously documented to be associated with a more severe injury and with more commonly neuromuscular deficits [5,7,18].

### Strengths and limitations of the study

The measurements used in this study are common among physical therapists and are recognised as easy to use, inexpensive, non-invasive and practical methods suitable for the present setting [8,9,19]. Although the methods used are subjective and not tested for validity or reliability in our population and inter-observer measurement bias cannot be ruled out [14,20-23], we believe that this possibility has been restricted by using two experienced physical therapists, focused training prior to the study, and a standardised protocol.

We acknowledge the limitations of the present study design. Although the majority of controls were from rural districts, they were recruited from the hospital area. They would therefore be more prone to have affected functions and be less representative for the background population. We speculate that more pronounced differences would have been observed with a control group recruited outside the hospital area. Further, some control patients recruited from outpatient departments refused to complete the whole assessment as it was time-consuming and they had to return a long way to their homes, they did not benefit from the study and the examination took valuable time from work and family. The time factor was difficult to account for, since we aimed at recruiting controls from outpatient departments rather than hospital wards. Such factors reduced the power of the study, especially when testing hip extension as it involved a change in testing position. Unfortunately we do not know whether these women were stronger or weaker than the ones remaining in the study. In order to strengthen statistics we refrained from more subtle changes and pooled observations into two groups, “full strength” and “less than full strength”. This was done to reduce the effect of subjective interpretation of degrees of strength. By analysing muscle groups instead of individual muscles, we may have missed subtle deficits of single muscles compensated by others. A more complete representation of the musculoskeletal deficits would have been achieved by including sensory and walk tests. However, du to time constrains these tests were not carried out.

## Conclusions

Women with obstetric fistula more commonly experienced leg pain and walking difficulties in the period after the injuring childbirth than the control group experienced after their childbirth. They presented with sequelae of significantly lower ankle joint range of motion and strength, lower range of knee joint motion but greater movement and strength in the hips, which might signify a compensatory adaptation. There seemed to be a potential for healing with time and some complete recovery even though the majority was left with a slightly altered test profile. This study brings attention to the altered musculoskeletal function in women with obstetric fistula years after the injury. The results of the present musculoskeletal study warrant a more detailed neurological assessment to fully appreciate the extent of the injury caused by the obstructed labour.

## Additional file

Additional file 1
**Strobe statement checklist of items that should be included in reports of** ***case-control studies.***
